# Sex-Specific Patterns of Asbestos Exposure and Prognostic Biomarkers in Surgically Treated Pleural Mesothelioma: A Retrospective Cohort Study

**DOI:** 10.3390/cancers18162624

**Published:** 2026-08-14

**Authors:** Nicolas Pinzon, Shubham Gulati, Andrew Del Re, Emanuela Taioli, Raja Flores, Andrea S. Wolf, Stephanie Tuminello

**Affiliations:** 1 Department of Thoracic Surgery, Icahn School of Medicine at Mount Sinai, New York, NY 10029, USA; nicolas.pinzon@icahn.mssm.edu (N.P.); shubham.gulati@icahn.mssm.edu (S.G.); emanuela.taioli@mountsinai.org (E.T.); raja.flores@mountsinai.org (R.F.); andrea.wolf@mountsinai.org (A.S.W.); 2 Institute for Translational Epidemiology, Icahn School of Medicine at Mount Sinai, New York, NY 10029, USA; 3 Tisch Cancer Center, Icahn School of Medicine at Mount Sinai, New York, NY 10029, USA

**Keywords:** pleural mesothelioma, sex disparities, asbestos, exposure history, PD-L1, Ki-67, BAP1, thoracic surgery

## Abstract

Background/Objectives: Sex-based differences in asbestos exposure and tumor biology in pleural mesothelioma (PM) remain incompletely characterized. As an expert tertiary center, we sought to systematically characterize asbestos exposure by sex and assess biomarker prognostic relevance in surgically treated pleural mesothelioma. Methods: We conducted a retrospective, single-center cohort encompassing all patients who underwent surgical intervention for PM at our institution. Demographic characteristics, asbestos exposure history, tumor biomarker profiles, and survival outcomes were extracted from this database and supplemented by review of the electronic medical record. Sex differences in asbestos exposure and biomarker expression were assessed using Fisher’s exact test on an available-case basis, while logistic regression was used to identify predictors of documented asbestos exposure. Overall survival was estimated using Kaplan–Meier analysis; independent associations between biomarkers and mortality were determined via multivariable Cox proportional hazards models adjusted for age, sex, and histological subtype. Results: Between 2015 and 2024, 106 patients (75% males) underwent surgical intervention for PM, with a median follow-up of 4.4 years. The mean age at surgery was 67.5 years (Standard Deviation [SD], 11). The cohort predominantly consisted of patients with epithelioid histology (*n* = 86, 81%), followed by biphasic (*n* = 16, 15%), and sarcomatoid (*n* = 4, 4%) subtypes. Thirty (28.3%) were alive at last follow-up. Median overall survival was 15.6 months (95% Confidence Interval [CI], 13.8–25.4 months), and 5-year survival was 17.8%. Of 92 patients with available exposure data, 66 (72%) had documented exposure histories. Although most of these exposures were occupational (46, 78%), a striking disparity was observed between occupational exposures in males vs. females (44 [86%] vs. 2 [25%]; *n* = 8 classifiable females; *p* < 0.001). Multivariable analysis demonstrated that male sex was associated with increased odds of asbestos exposure (Adjusted Odds Ratio [OR_adj_], 5.45; 95% Confidence Interval [CI], 1.90–16.35). PD-L1, Ki-67, or BAP1 expression were not associated with sex, age, or asbestos exposure. High Ki-67 (>15%) and PD-L1 (>15%) were associated with a four-fold and three-fold increased risk of mortality (Hazard Ratio_adj_ [HR_adj_], 4.33 [95% CI, 1.85–10.13] and HR_adj_, 2.80 [95% CI, 1.39–5.64], respectively), whereas BAP1 loss was not (HR_adj_, 0.80; 95% CI, 0.45–1.44). Conclusions: Our findings underscore the potential value of sex-sensitive screening protocols that more comprehensively assess non-occupational asbestos exposure pathways and offer additional evidence regarding the prognostic relevance of PD-L1, Ki-67, and BAP1 in surgical patients.

## Simple Summary

Pleural mesothelioma (PM) is an aggressive cancer primarily caused by asbestos exposure. While historically described to affect males working in industrial settings, females are often exposed through non-occupational pathways. We analyzed data from 106 surgically treated PM patients to better understand exposure patterns by sex and evaluate how three specific tumor biomarkers (PD-L1, Ki-67, and BAP1) affect survival. We found a major exposure disparity by sex: 86% of exposed males had documented occupational histories, compared to only 25% of females (*n* = 8 classifiable females). Furthermore, female exposure histories were more often classified as “unknown”. These findings suggest that routine exposure assessment may insufficiently capture non-occupational asbestos exposure routes in females. Survival analyses revealed that patients with high PD-L1 and Ki-67 expression (>15%) experienced significantly shorter survival. Conversely, BAP1 loss did not affect survival. These findings help identify high-risk patients who may benefit most from multi-modal therapy.

## 1. Introduction

Pleural mesothelioma (PM) is a rare and aggressive malignancy arising from the mesothelial lining of the pleura, with an annual incidence of approximately 1 per 100,000 in the United States and a 5-year survival rate of 5–10% [1,2]. Asbestos exposure is the primary and most well-established cause of PM. Since the seminal case series that first reported this link in the 1960s, our understanding of the disease has evolved from a pathological curiosity to a preventable occupational disease with a characteristically long latency period of 20 to 50 years between exposure and diagnosis [1,3,4]. Although historically defined by male-dominated industrial cohorts, data suggest that occupational asbestos exposure accounts for approximately 83% of mesothelioma cases in males, compared to only 42% in females [5]. Prominently, non-occupational exposure plays a significantly larger role for females, attributing to 39% of cases in this group and bringing the total attributable risk in this population to 65% [5]. Notably, this distinct epidemiological profile is accompanied by a robust female survival advantage, consistently documented across several large-scale database analyses [6,7] and shown to persist independent of age, histological subtype, and performance status [8]. Yet, despite these divergent clinical and exposure patterns, female exposure histories remain poorly documented in the literature, with profound implications for understanding disease etiology and biology, supporting compensation claims, and informing public health policy [9]. Given the significant burden of non-occupational disease in females, it is of great interest to systematically report their exposure histories and continue to bridge this epidemiological gap.

Beyond epidemiological characterization, a critical focus of modern oncological research is the development of prognostic biomarkers to guide risk stratification, treatment intensity, and patient counseling. However, the identification and validation of such prognostic biomarkers remains a formidable challenge [10]. In fact, fewer than 1% of published candidates ultimately transition into clinical practice [11]. For pleural mesothelioma (PM), these difficulties are compounded by the disease’s inherent histomorphologic and molecular heterogeneity, with biomarker significance varying substantially across the three primary histologic subtypes: epithelioid, biphasic, and sarcomatoid [12]. These subtypes fundamentally dictate the clinical trajectory; notably, the latest ASCO guidelines report a median survival of 19, 12, and 4 months for epithelioid, biphasic, and sarcomatoid PM, respectively [13].

In parallel with the aforementioned epidemiological disparities, the prognostic landscape of key tumor biomarkers in PM remains fragmented. Currently, Programmed Death-Ligand 1 (PD-L1) expression and the Ki-67 proliferation labeling index are the only markers consistently positively associated with mortality across treatment modalities, though their prognostic value has been demonstrated in cohorts with predominantly epithelioid subtype [12,14]. However, data characterizing the association between these biomarkers and sex remains sparse. PD-L1 has one reported null finding by sex [15], while there are no sex-stratified data in PM for Ki-67. Conversely, despite the growing clinical focus on BRCA1-associated protein 1 (BAP1), its prognostic significance remains inconsistent and highly dependent on clinical context. While data from chemotherapy-treated cohorts suggest BAP1 loss may predict a better response to platinum-pemetrexed chemotherapy regimens, meta-analyses of mixed populations have failed to establish it as an independent prognostic factor [16,17]. The strongest molecular link to sex identified to date is through germline predisposition, with the latest ASCO guidelines reporting that germline BAP1 pathogenic/likely pathogenic variant carriers are enriched for female sex [13]. Critically, data regarding BAP1 in surgically treated patients remains sparse. As BAP1 is the most frequent mutation in PM, there is an urgent need for evaluation within specific treatment subgroups [18]. Validating the prognostic capability of these biomarkers specifically within surgical cohorts is therefore essential to refining clinical decision-making.

As a high-volume academic tertiary center with expertise in PM, we sought to leverage our surgically treated cohort to address two distinct but complementary aims: to systematically characterize asbestos exposure and biomarker presence by sex, and to evaluate the association of these biomarkers with survival outcomes within the same well-characterized surgical population.

## 2. Materials and Methods

### 2.1. Study Design

We conducted a single-center retrospective cohort study at the Icahn School of Medicine at Mount Sinai (New York, NY, USA), an expert tertiary referral center for thoracic malignancies. This observational study was reported in accordance with the Strengthening the Reporting of Observational Studies in Epidemiology (STROBE) guidelines for cohort/case–control/cross-sectional studies.

### 2.2. Setting

The Mount Sinai Health System, located in New York, NY, USA, is a tertiary referral health system with specialized expertise in the diagnosis and treatment of thoracic malignancies. All cases were identified through the Department of Thoracic Surgery’s prospectively maintained database, which records diagnosis, procedure type, and all other relevant clinical variables. Data were collected from 1 January 2015 to 31 December 2024; patients were followed until death or last contact, with the follow-up cut-off date set at 15 January 2026.

### 2.3. Participants

We included all consecutive patients who underwent surgical intervention for pleural mesothelioma between 1 January 2015, and 31 December 2024. Surgical interventions included pleurectomy/decortication (P/D), extended pleurectomy/decortication (eP/D), and partial pleurectomy, as reported by the operating surgeon at the time of database entry. To ensure a comprehensive analysis of the surgical population, no exclusion criteria were applied. A total of 106 patients met inclusion criteria and were included in the final analytic cohort.

### 2.4. Data Sources

Patients for this study were identified using the Department of Thoracic Surgery’s prospectively maintained database, which contained data on patient demographics and clinical characteristics. Asbestos exposure histories were manually abstracted from the electronic medical records (EMR) by trained researchers (NP, SG). Biomarker profiles were extracted from institutional pathology reports derived from surgical resection specimens, pre-operative biopsies, and cytopathology of pleural fluid.

### 2.5. Variables

The primary outcome was overall survival. The following predictors were considered: age (as continuous), sex (male, female), histotype (epithelioid, sarcomatoid, biphasic), the asbestos exposure (occupational, non-occupational, no exposure, or unknown/missing), and tumor biomarkers such as PD-L1 expression (grouped as ≤15% or >15%), Ki-67 labeling index (grouped as ≤15% or >15%), and BAP1 status (intact or lost, based on nuclear staining).

#### 2.5.1. Asbestos Exposure

After comprehensive EMR review, a “likely asbestos exposure” was defined as a documented history of: (1) direct occupational exposure (e.g., construction, shipyard, factory, or electrical work); (2) secondary exposure via an exposed household member or marital partner; (3) environmental exposure (confirmed presence of asbestos in the primary residence); (4) childhood exposure; or (5) unknown reported asbestos exposure combined with a longstanding occupational history where asbestos was common. In the absence of these specific mentions, patients who explicitly reported no asbestos exposure were categorized as none, while those without documentation of exposure history were classified as unknown. Subsequently, an occupational exposure history was defined as documented history of either category (1) or (5) as reported above. Categories (2), (3), and (4) were classified as non-occupational exposure pathways.

Because category (5) relies on an inferred occupational history rather than a directly documented exposure, accounting for 7 of the 45 men (15.6%) classified as occupationally exposed, we performed a sensitivity analysis excluding these patients (Supplementary Tables S1 and S2). Additionally, because asbestos exposure history was missing or unknown for 14 of 106 patients (13.2%), we performed a sensitivity analysis comparing age, sex, and histological subtype between patients with documented versus missing/unknown exposure data, to assess whether this missingness was likely to introduce systematic bias (Supplementary Table S3).

#### 2.5.2. Tumor Biomarkers

Biomarker profiles were extracted from institutional pathology reports derived from surgical resection specimens, pre-operative biopsies, and cytopathology of pleural fluid. The primary analysis focused on three biomarkers with established or emerging prognostic significance: PD-L1, Ki-67, and BAP1. PD-L1 expression was assessed using the Dako PD-L1 IHC 22C3 PharmDx immunohistochemistry assay, with results reported according to the tumor proportion score (TPS). Ki-67 and BAP1 expression were assessed by immunohistochemistry as part of routine clinical pathology, and BAP1 nuclear staining was classified as retained or lost expression, while Ki-67 labeling index was recorded as the percentage of positively stained tumor cell nuclei. For quantitative analysis, PD-L1 expression and the Ki-67 labeling index were dichotomized using a cut-off of 15% (grouped as ≤15% or >15%). This threshold is well-established for Ki-67 in PM [12,19]. For PD-L1, mesothelioma-specific evidence for a 15% threshold is more limited, drawn from a single prior study [20]. Given that the literature also reports 1%, 5%, and 50% thresholds, we additionally repeated the PD-L1 analyses using alternative reported cutoffs (>1% and ≥50%) as a sensitivity analysis (Supplementary Table S4 and Figure S1).

Because biomarker testing was subject to variable missingness, (BAP1: *n* = 25, 23.6%; PD-L1: *n* = 38, 35.8%; Ki-67: *n* = 57, 53.8%), we performed a sensitivity analysis comparing age, sex, histological subtype, and overall survival between patients with and without available data for each biomarker, to assess whether this missingness was likely to introduce systematic bias into our survival analyses (Supplementary Tables S5–S7). Additionally, because histological subtype is a potential confounder of biomarker–survival associations, we performed a sensitivity analysis restricting our multivariable Cox proportional hazards models to patients with epithelioid histology (*n* = 86), to assess whether the biomarker associations with overall survival remained consistent within this histologically homogeneous subgroup (Supplementary Table S8).

#### 2.5.3. Follow up

The primary outcome was overall survival, defined as the interval from the date of surgery to death from any cause. Survival status and dates of death were obtained from the institutional EMR. To improve mortality ascertainment and minimize loss to follow-up, EMR data were supplemented by systematic searches of publicly available obituary records. Linkage to a population-based cancer registry or the National Death Index was not performed because the associated fees, permissions, and administrative requirements were beyond the scope of this unfunded, single-center retrospective study. Patients without a documented death were censored at the date of their last recorded clinical encounter. Final follow-up data were collected on 15 January 2026. Median follow-up was estimated using the reverse Kaplan–Meier method.

### 2.6. Data Quality

Data quality was considered with respect to provenance, completeness, consistency, and fitness for the intended analyses, in accordance with established frameworks for the secondary use of EMR data [21,22]. Because the EMR was created primarily for clinical care rather than research, the completeness of individual variables depended on the quality of clinical documentation, patient recall, tissue availability, testing practices, healthcare utilization, and whether care was received within our health system.

Asbestos exposure was retrospectively classified using a predefined five-category framework outlined in Section 2.5.1. Nevertheless, household, childhood, environmental, and other indirect exposures may have been incompletely recalled or documented, and occupation-associated exposure could not always be independently verified. We therefore performed a sensitivity analysis excluding patients classified as occupationally exposed solely on the basis of an occupation in which asbestos exposure was historically common (category 5). The association between male sex and likely asbestos exposure remained significant after these patients were excluded (Supplementary Tables S1 and S2).

Biomarker results were obtained from heterogeneous specimen sources, including surgical resection specimens, preoperative biopsies, and pleural-fluid cytopathology. Differences in specimen type, sampling, tissue adequacy, and timing of collection may therefore have introduced measurement variability. Biomarker testing was performed during routine clinical care rather than according to a uniform prospective protocol. BAP1, PD-L1, and Ki-67 results were unavailable for 25 (23.6%), 38 (35.8%), and 57 (53.8%) patients, respectively. Comparisons between patients with and without available data demonstrated no statistically significant differences in age, sex, histological subtype, or overall survival for any of the three biomarkers (Supplementary Tables S5–S7).

Mortality status was obtained from the EMR and supplemented with publicly available obituary records. Population-based cancer registry and National Death Index linkage were not performed because the associated costs and administrative requirements were beyond the scope of this unfunded study. Although supplementation with external data improves mortality ascertainment compared with reliance on structured EMR data alone [23], deaths occurring outside our healthcare system may still have been missed, particularly among patients who relocated, changed healthcare providers, or no longer received care within the institution.

### 2.7. Statistical Analysis

Categorical variables were presented as frequencies and percentages, while continuous variables were reported as means with standard deviations (SD) or medians with interquartile ranges (IQR). Baseline differences between male and female patients were assessed using two-sample *t*-tests for age and Fisher’s exact tests for asbestos exposure history and biomarker expression (BAP1, PD-L1, and Ki-67) via the compareGroups R package. To evaluate independent predictors of asbestos exposure, univariable and multivariable logistic regression models were constructed, with results reported as odds ratios (OR) and adjusted odds ratios (OR_adj_) with 95% confidence intervals (CI).

The primary outcome, overall survival, was estimated using the Kaplan–Meier method, and differences between groups were evaluated using the log-rank test. To determine the independent prognostic value of tumor biomarkers, separate multivariable Cox proportional hazards regression models were constructed for each biomarker. These models were adjusted for age, sex, and histological subtype as potential confounders. For the BAP1 model, intact expression served as the reference; for PD-L1 and Ki-67, expression ≤ 15% was used as the reference. Survival outcomes were reported as adjusted hazard ratios (HR_adj_) with 95% CIs. The proportional hazards assumption was verified using Schoenfeld residuals. Because each biomarker was modeled separately, this approach does not permit assessment of whether the associations of BAP1, PD-L1, and Ki-67 with overall survival are independent of one another. Consequently, the relative contribution of each biomarker could not be determined due to the small sample size.

Analyses were performed on an available-case basis. Multiple imputation was not performed because biomarker availability was primarily determined by whether testing had been performed during routine clinical care. The dataset lacked sufficiently informative auxiliary variables to support reliable imputation, particularly for Ki-67, for which more than half of observations were unavailable. To evaluate the potential effect of missingness, we compared age, sex, histological subtype, and overall survival between patients with and without available data for each biomarker (Supplementary Tables S5–S7). Little’s test was also performed to evaluate whether the overall pattern of missingness provided evidence against the assumption that the data were missing completely at random. The test did not provide evidence against this assumption (χ^2^ = 52.8, df = 49, *p* = 0.330), although this result cannot exclude more complex patterns of missingness related to unmeasured factors. Of the 106 patients, missing data were as follows: asbestos exposure (*n* = 14), BAP1 expression (*n* = 25), PD-L1 expression (*n* = 38), and Ki-67 labeling index (*n* = 57). All statistical tests were two-sided, and a *p*-value of less than 0.05 was considered significant. Statistical analyses were conducted using R version 4.5.2.

## 3. Results

### 3.1. Study Cohort Characteristics

Between 2015 and 2024, a total of 106 patients underwent surgical intervention for pleural mesothelioma. The cohort was predominantly male (*n* = 79 [75%]), with a mean age at surgery of 67.5 years (SD, 11). Histological distribution was primarily epithelioid (*n* = 86 [81%]), followed by biphasic (*n* = 16 [15%]) and sarcomatoid (*n* = 4 [4%]) subtypes (Table 1). At the final data cutoff on 15 January 2026, 30 patients (28.3%) were alive. The median overall survival for the entire cohort was 15.6 months (95% CI 13.8–25.4 months), and the 5-year survival was 17.8% (*n* = 7 patients remaining at risk at 5 years). Median follow-up for the cohort was 4.4 years (95% CI, 2.6 years—not reached).

### 3.2. Asbestos Exposure by Sex

Of the 92 patients with available exposure data, 66 (72%) had a likely asbestos exposure, with a significant sex-based disparity in asbestos exposure histories (Table 1). Among the 70 male patients with available exposure data, 57 (81%) had a likely asbestos exposure. In contrast, among the 22 female patients with available exposure data, only 9 (41%) had a likely history of asbestos exposure (*p* < 0.001). Of the 66 patients with a likely asbestos exposure history, we further classified the source of exposure for 59 (51 males and 8 females). There were 44 (86%) exposed males linked to a documented occupational pathway, while only 2 (25%) of exposed females were (*p* < 0.001).

In univariable analysis, male sex was associated with a six-fold increase in the odds of likely asbestos exposure (OR 6.33; 95% CI 2.28–18.60). Additionally, increasing age was associated with higher odds of exposure (OR 1.04 per year; 95% CI 1.01–1.09) (Table 2). In multivariable analyses, male sex remained independently associated with likely asbestos exposure (OR_adj_ 5.45; 95% CI 1.90–16.35), while the association with increasing age was attenuated and no longer reached statistical significance (OR_adj_ 1.04; 95% CI 0.99–1.08). To assess whether this disparity was driven by the inferred occupational-exposure category (category 5), we repeated these analyses after excluding the 7 patients whose occupational exposure was based on this criterion (Supplementary Tables S1 and S2). The disparity persisted: likely asbestos exposure remained strongly associated with male sex (40.9% of women vs. 79.4% of men, *p* = 0.002), as did occupational exposure specifically (25.0% of women vs. 86.4% of men, *p* = 0.001). In logistic regression, the univariable odds ratio for male sex attenuated only modestly, from 6.33 (95% CI 2.28–18.60) in the full cohort to 5.56 (95% CI 1.99–16.38) excluding category (5), and the multivariable, age-adjusted estimate likewise moved only slightly, from 5.45 (95% CI 1.90–16.35) to 4.99 (95% CI 1.74–14.92; *p* = 0.003).

### 3.3. Biomarker Expression

Biomarker data were available preoperatively (from preoperative biopsies and cytopathology of pleural fluid) for 68 (64%) of patients, and postoperatively (from surgical resection specimens) for 38 (36%) of patients. Of the patients with preoperative data, 29 (43%) had both a biopsy and cytopathology available, 27 (40%), had a biopsy only, and 12 (17%) had cytopathology only. Due to variation in tissue availability and molecular testing, data were available for 81 patients for BAP1, 68 for PD-L1, and 49 for Ki-67.

BAP1 loss was identified in 46 (57%) of 81 patients. Patients with loss of BAP1 expression were significantly older (mean 69.5 years [SD 8.32]) than those with retained BAP1 (64.3 years [SD 13.6]; *p* = 0.049), though no statistically significant associations were observed with sex (*p* = 1.000) or asbestos exposure (*p* = 0.107). High Ki-67 expression (>15%) was observed in 30 (61%) of 49 patients. Ki-67 status was not significantly associated with sex (*p* = 1.000), age (*p* = 0.854), or asbestos exposure (*p* = 0.501). PD-L1 expression > 15% was found in 18 (26%) of 68 patients. While PD-L1 expression > 15% showed a marginal trend toward association with female sex (43% of females vs. 19% of males; *p* = 0.080) and a lack of asbestos exposure (44.4% vs. 20.5%; *p* = 0.067), these did not reach statistical significance (Table 1).

### 3.4. Survival Analysis

**Figure 1:**
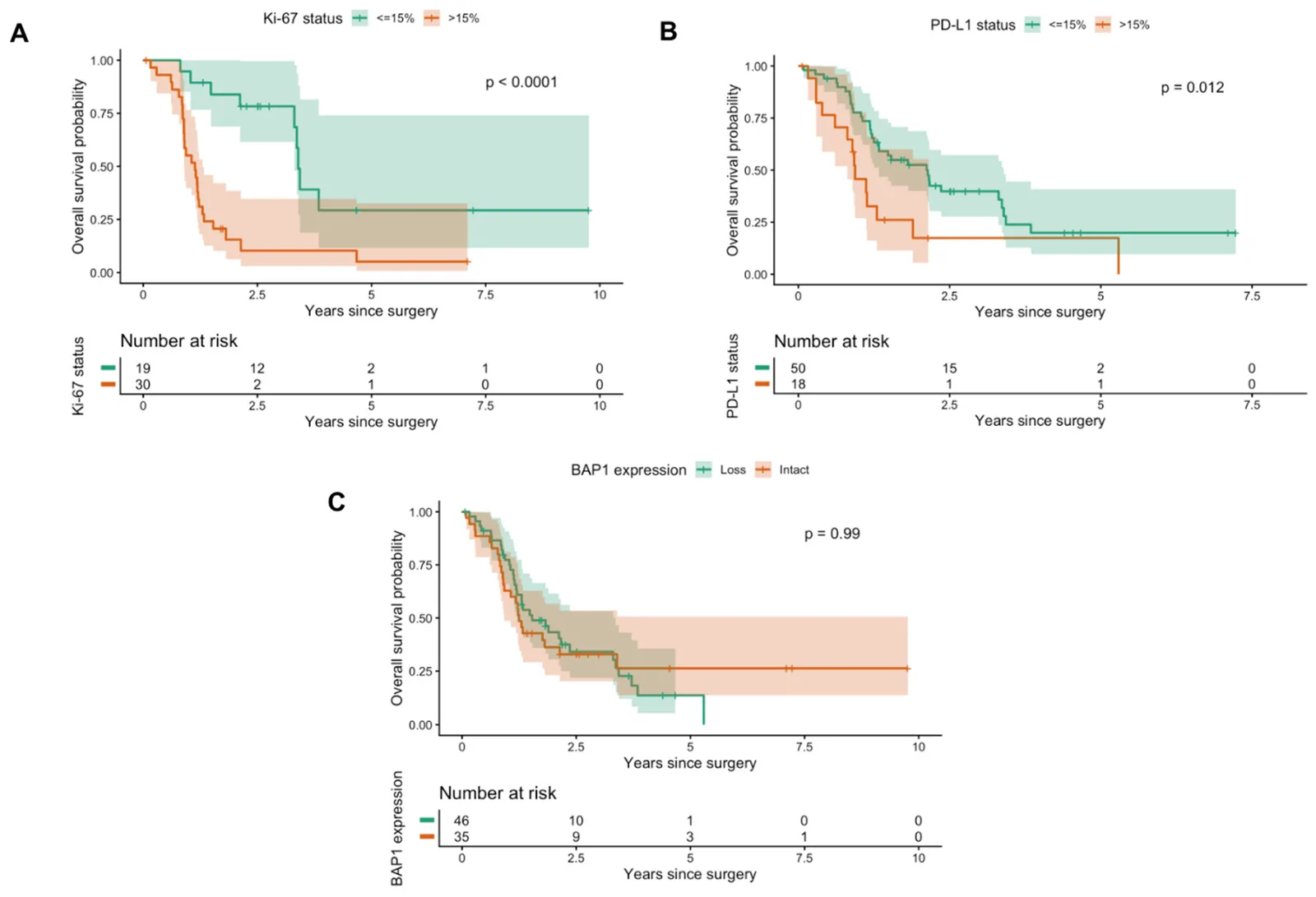
Overall survival of patients undergoing surgical intervention for pleural mesothelioma stratified by biomarker status. Kaplan–Meier survival curves illustrating overall survival according to (**A**) Ki-67 labeling index (≤15% vs. >15%), (**B**) PD-L1 tumor proportion score (≤15% vs. >15%), and (**C**) BAP1 expression (intact vs. loss). Survival data were obtained from electronic medical records and supplemented with publicly available obituary data to minimize loss to follow-up through 15 January 2026. Shaded areas represent 95% confidence intervals. A log-rank test was performed to determine *p*-values and significance. The number of patients at risk is provided below each panel. Note: Denominators vary between panels due to the availability of biomarker data (*n* = 81 for BAP1, *n* = 68 for PD-L1, and *n* = 49 for Ki-67).

**Figure 2:**
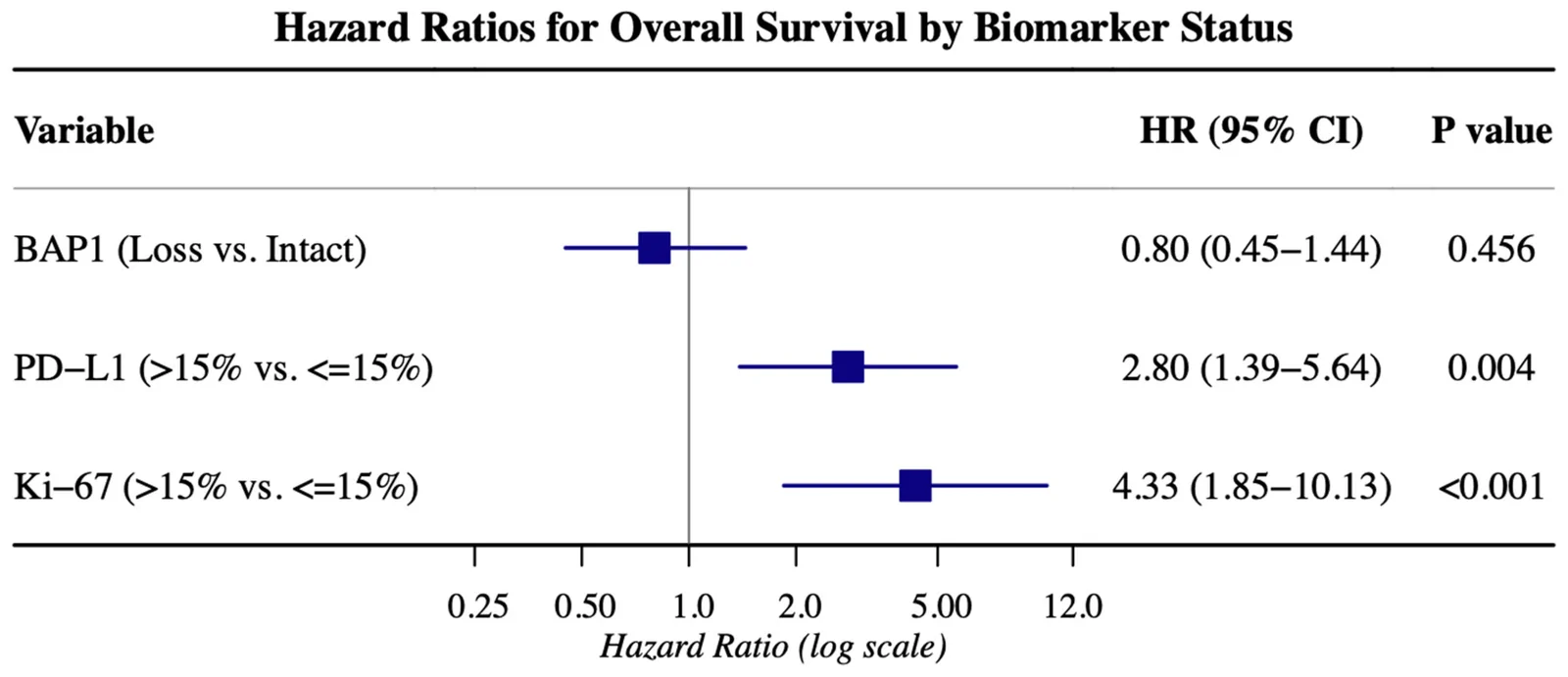
Multivariable Cox proportional hazards models for overall survival in patients undergoing surgical intervention for pleural mesothelioma. Forest plot displaying adjusted hazard ratios (HR_adj_) for mortality. Separate multivariable models were constructed for BAP1 status (loss vs. intact), PD-L1 expression (>15% vs. ≤15%), and Ki-67 labeling index (>15% vs. ≤15%). Each model was adjusted for age, sex, and histological subtype to determine independent associations with survival. The vertical line represents an HR_adj_ of 1.0 (null hypothesis). Horizontal lines represent 95% confidence intervals (CI). An HR_adj_ > 1.0 indicates an increased risk of mortality. Note: Sample sizes vary by model due to the availability of biomarker data: BAP1 (*n* = 81, 57 events), PD-L1 (*n* = 68, 47 events), and Ki-67 (*n* = 49, 35 events).

In multivariable Cox proportional hazard models adjusting for age, sex, and histological subtype stratified by biomarker, we found PD-L1 and Ki-67 expression to be independent predictors of mortality. High Ki-67 expression (>15%) was associated with the greatest risk of death (HR_adj_ 4.33; 95% CI 1.85–10.13; *p* < 0.001), followed by PD-L1 expression > 15% (HR_adj_ 2.80; 95% CI 1.39–5.64; *p* = 0.004). In contrast, we did not find BAP1 loss to be statistically significantly associated with overall survival (HR_adj_ 0.80; 95% CI 0.45–1.44; *p* = 0.456) (Figure 1 and Figure 2). Sensitivity analyses using alternative PD-L1 cutoffs showed that the association between elevated PD-L1 expression and overall survival was consistent in direction and magnitude at both the primary > 15% threshold and the ≥50% threshold (HR_adj_ 2.58, 95% CI 1.18–5.66, *p* = 0.018). At a minimal positivity threshold of >1%, the point estimate remained in the same direction but the association attenuated and did not reach statistical significance (HR_adj_ 1.47, 95% CI 0.75–2.87, *p* = 0.263) (Supplementary Table S4 and Figure S1). Notably, Ki-67 data were available for only 49 of the 106 patients (46%), the smallest subgroup among the three biomarkers evaluated; our results should be interpreted in light of this. Because histological subtype is a potential confounder of biomarker–survival associations, we additionally performed a sensitivity analysis restricting each multivariable Cox model to patients with epithelioid histology (*n* = 86) (Supplementary Table S8). In this epithelioid-only subset, both Ki-67 (HR_adj_ 5.35; 95% CI 2.05–13.98; *p* < 0.001) and PD-L1 (HR_adj_ 3.81; 95% CI 1.71–8.47; *p* < 0.001) remained significant predictors of mortality. Additionally, BAP1 remained non-significant (HR_adj_ 1.08; 95% CI 0.57–2.04; *p* = 0.814).

## 4. Discussion

In this retrospective cohort study of 106 surgically treated PM patients, we report three principal findings. First, we demonstrate a significant sex-based disparity in documented asbestos exposure histories, with male patients having five-fold increased odds of likely exposure compared to females. This finding strongly underscores the continued gap in understanding of non-occupational exposure pathways in females. Second, we describe PD-L1 and Ki-67 expression as independent predictors of mortality in surgically treated patients, with high Ki-67 (>15%) conferring a four-fold increased risk of death and PD-L1 (>15%) a three-fold increased risk. Third, we observe that BAP1 loss is not associated with overall survival in this surgical cohort, adding important treatment-specific data to a largely inconsistent body of literature that has predominantly evaluated BAP1 in chemotherapy-treated populations. The survival outcomes, median overall survival of 15.6 months and 5-year survival of 17.8%, are consistent with published surgical series, supporting the aggressive nature of this malignancy even in patients carefully selected for surgical intervention [1,13,24]. Together, these findings clarify underreported sex-based differences in documented asbestos exposure histories and refine the prognostic relevance of key biomarkers specifically for surgically treated pleural mesothelioma.

Our findings that male patients had five-fold increased odds of likely asbestos exposure compared to females are consistent with the well-documented sex-based disparity in asbestos exposure pathways [5]. This disparity has been further documented in other populations: Australian and Danish studies have reported occupational histories in only 6.8% and 9% of female cases, respectively [25,26]. Similarly, an analysis of the Italian National Mesothelioma Registry found that the female-to-male ratio dropped from 0.38 to 0.14 when restricted to occupationally exposed cases [27]. Our granular EMR review further quantifies this gap. In this large mesothelioma cohort in a population with high exposure-awareness, we found that among patients with a classifiable exposure source, 86% of males were linked to direct occupational pathways, whereas only 14% of females were exposed via this pathway. Critically, female exposure histories are most frequently reported as “unknown” or fail to identify a specific exposure source [28]. These findings raise the possibility that non-occupational exposures among females are systematically under ascertained, especially given that studies have found that female mesothelioma cases cluster in the same high-risk geographic areas as male cases [29]. The clinical implications of this identification gap are substantial, as the incidence of mesothelioma in males has substantially declined since the early 2000s, but incidence in females has remained relatively stable, or at the very least, declined more slowly [25,30]. This divergence suggests that non-occupational exposure pathways continue to drive disease in females, perhaps because such pathways are harder to identify and eliminate. Nevertheless, these data highlight the potential value of systematic, standardized, and sex-sensitive exposure assessment protocols that explicitly assess non-occupational asbestos, which may be incompletely captured during routine clinical history-taking.

Our finding that BAP1 loss is not associated with sex and with overall survival in this surgical cohort (HR 0.80; 95% CI 0.45–1.44) adds important treatment-specific data to a fragmented and largely inconsistent body of literature. While BAP1 is the most frequently altered gene in PM, present in 50–70% of epithelioid cases, its prognostic significance remains a subject of ongoing debate [18,31]. The strongest evidence for BAP1’s clinical utility comes from chemotherapy-treated populations: a validation study across Danish and Australian cohorts, which explicitly excluded surgical cases, demonstrated that BAP1 loss predicted better survival with first-line platinum-pemetrexed (median survival: 20.1 vs. 7.3 months and 19.6 vs. 11.1 months, respectively) [16]. Patients with retained BAP1 derived no survival benefit from chemotherapy compared to best supportive care alone, whereas patients with BAP1 loss experienced substantially better survival with chemotherapy, suggesting BAP1 may function as a predictive marker of chemotherapy benefit rather than a purely prognostic factor [16]. In contrast, meta-analyses of mixed treatment populations have failed to establish BAP1 as an independent prognostic factor (HR 1.11; 95% CI 0.76–1.61), with authors concluding that BAP1 should not be considered without accounting for tumor histotype and treatment context [17]. Our null finding in a surgical cohort is consistent with this treatment-specific framework: if BAP1’s prognostic capability derives primarily from its association with chemotherapy response, it would not be expected to predict outcomes in patients whose primary treatment modality is surgical resection. However, because many surgically treated patients also receive systemic therapy, this interpretation should be considered hypothesis-generating rather than definitive. These findings underscore the need for continued investigation of BAP1 across treatment-specific subgroups, consistent with recent reviews concluding that BAP1 is not yet suitable for clinical practice as a prognostic or predictive factor [18]. Accordingly, our findings should be interpreted in the context of a surgically treated population and should not be extrapolated to patients managed with chemotherapy alone or other non-surgical approaches, in whom BAP1’s prognostic utility may differ.

In contrast to the inconsistent findings for BAP1, our PD-L1 and Ki-67 data align with previously reported findings in the literature, and independently confirm that these two biomarkers are robust independent predictors of mortality in surgically treated PM. For Ki-67, a multicenter study of 285 patients demonstrated that Ki-67 > 15% was independently prognostic in epithelioid PM (HR 2.1; *p* < 0.001), with patients having high Ki-67 experiencing median survival of only 7.5 months compared to 19.1 months for those with low Ki-67 [10]. More recently, a 2024 multicenter analysis specifically evaluating surgical cohorts found that Ki-67 ≤ 15% was associated with median survival of 31.2 months compared to 11.1 months for Ki-67 > 15%, with 5-year survival of 22% versus 5%, respectively [19]. Notably, this study identified Ki-67 as not only prognostic but also predictive of treatment benefit from surgery within multimodality therapy, suggesting that Ki-67 may help identify patients most likely to benefit from surgical intervention [19]. For PD-L1, meta-analyses comprising over 1600 patients have consistently demonstrated that elevated PD-L1 expression predicts lower overall survival (pooled HR 1.50; 95% CI 1.32–1.70), irrespective of cutoff value or ethnicity [14]. Importantly, a study specifically evaluating patients undergoing macroscopic complete resection found that high PD-L1 expression (≥50%) was independently associated with poor overall survival (HR 5.67; *p* < 0.001) and poor recurrence-free survival (HR 3.28; *p* = 0.003), concluding that PD-L1 may be useful in perioperative decision-making [32]. In our cohort, sensitivity analyses demonstrated a consistent, statistically significant prognostic association at both the >15% and ≥50% thresholds (HR_adj_ = 2.80 and 2.58, respectively). At a minimal >1% threshold, the point estimate remained directionally consistent (HR_adj_ = 1.47) but did not reach statistical significance (Supplementary Table S4 and Figure S1). Collectively, our findings extend previous evidence by confirming the prognostic value of both markers specifically within a surgical cohort using a >15% cutoff, providing treatment-specific validation that strengthens the case for incorporating these biomarkers into risk stratification, ensuring that highest-risk patients receive complementary therapies alongside surgery.

The combination of sex-based exposure disparities and molecular prognosticators suggests a dual-track approach to improving PM outcomes. While the epidemiological gap in females necessitates more nuanced clinical history-taking to identify non-occupational exposures, the biological data provide a clear roadmap for surgical selection. Our findings suggest that evaluating PD-L1 and Ki-67 should be prioritized when counseling patients on their long-term prognosis and the potential necessity of supplemental multimodal therapies. Patients with Ki-67 or PD-L1 expression > 15% may represent a high-risk subgroup with poorer postoperative outcomes. If validated in larger cohorts, these biomarkers could have value for preoperative risk stratification and sequencing. Further, they may warrant additional investigation as tools to inform multidisciplinary treatment planning, including neoadjuvant immunotherapy, clinical trial enrollment, and multimodal treatment approaches. Due to sample size limitations, we did not formally test whether the prognostic impact of PD-L1, Ki-67, and BAP1 differs by sex. We strongly encourage future projects reliant on larger databases to address this unmet but clinically relevant research gap.

### 4.1. Limitations

This study has several limitations. First, it was conducted at a single high-volume tertiary referral center and included only patients selected for surgical intervention. These patients may differ systematically from the broader pleural mesothelioma population in disease stage, performance status, comorbidity burden, referral patterns, treatment access, and socioeconomic characteristics. The resulting referral and surgical-selection biases limit the generalizability of our findings to patients managed nonoperatively or treated in other clinical settings.

Second, given that our cohort had a median follow-up time of 4.4 years, 5-year overall survival estimates rely on a progressively smaller number of patients remaining at risk beyond the median follow-up (*n* = 7 at 5 years) and should therefore be interpreted with appropriate caution. Additionally, given that mortality status was determined using the EMR supplemented by obituary records and was not independently verified through a population-based cancer registry or the National Death Index, deaths occurring outside our healthcare system may have been missed. Lastly, demographic, exposure, and clinical variables were originally collected for clinical rather than research purposes. Consequently, the completeness and reliability of these variables depended on the quality of documentation, patient recall, tissue availability, testing practices, and care received within our healthcare system. These limitations are particularly relevant to asbestos exposure histories, for which indirect, household, childhood, and environmental exposures may have been incompletely documented or misclassified.

Third, while our total cohort consisted of 106 patients, missing data were present for both asbestos exposure (*n* = 14, 13.2%) and biomarker testing, particularly for Ki-67 (*n* = 57, 53.8%). To assess whether this missingness introduced systematic bias, we compared age, sex, histological subtype, and (for biomarkers) overall survival between patients with and without available data; no significant differences were observed in any comparison (Supplementary Tables S4–S7). Little’s test further did not provide evidence against the assumption that these data are missing completely at random (χ^2^ = 52.8, df = 49, *p* = 0.330), though this test cannot rule out more complex patterns of missingness. Although biomarker subgroups were sufficient to demonstrate high statistical significance in multivariable models, the reduced sample size may limit the generalizability of the point estimates. While our multivariable models successfully adjusted for histological subtype, the relatively small number of non-epithelioid cases (*n* = 20) may limit our ability to detect subtype-specific variations in biomarker performance.

Additionally, our multivariable models did not account for other potentially confounding variables not assessed in our study, such as disease stage, extent of surgery, treatment regimen, performance status, comorbidities, lifestyle factors, and socioeconomic status. Our results should be interpreted in light of this limitation. Future multi-institutional studies are needed to confirm these findings accounting for such other factors and including larger cohorts of biphasic and sarcomatoid patients undergoing surgery. In addition, we did not formally test whether the prognostic impact of PD-L1, Ki-67, and BAP1 differs by sex, as the resulting subgroups were too small to support a reliable interaction analysis. Larger, multi-institutional studies with greater representation of female patients are needed to investigate sex-specific findings and improve the generalizability of our results.

Finally, while our study highlights a significant sex-based disparity in exposure and exposure pathways, we acknowledge the inherent challenges in classifying non-occupational asbestos exposure. Asbestos exposure was retrospectively ascertained through EMR review and depended on patient recall and the completeness of clinical documentation. Non-occupational exposure pathways, including household, childhood, and environmental exposures, may have been underreported or misclassified, while some occupation-associated exposures could not be independently confirmed. Although the sex-based association remained significant after excluding the inferred occupational-exposure category, residual exposure misclassification remains possible. Additionally, because female exposure pathways are often indirect or environmental, they lack the ‘definitive’ documentation typical of male-dominated industrial trades. While we employed a stringent and granular EMR review strategy to mitigate this, the qualitative nature of these histories introduces a degree of classification uncertainty. Nevertheless, we argue that such detailed manual review is necessary to move beyond the systematic under-reporting of female exposures that remains a pervasive challenge in mesothelioma epidemiology.

### 4.2. Strengths

Despite these limitations, this study has several strengths. First, while a cohort of 106 patients may appear modest in other oncological research contexts, it represents a substantial surgical case series for PM, particularly within a single-institution framework. For comparison, recent single-institution surgical series have reported cohorts of 62 to 163 patients accumulated over 10 to 20 years, and the largest randomized surgical trial in PM (MARS2) enrolled 335 patients across multiple centers over a similar period [33,34,35,36,37]. As an expert, high-volume tertiary referral center, our cohort represents a specialized surgical population treated with consistent institutional protocols and multidisciplinary expertise. Second, our enhanced EMR methodological approach improves the granularity and completeness of this analysis beyond what is typically available in retrospective studies. By supplementing EMR data with systematic searches of publicly available obituary records, we minimized loss to follow-up for survival outcomes. Additionally, we manually parsed through each patient’s EMR to classify their asbestos exposure, employing a five-category framework that distinguishes occupational, second-hand, environmental, childhood, and occupation-associated pathways of exposure. Such granularity is essential for uncovering the non-occupational pathways that disproportionately affect female patients. Lastly, our cohort includes 27 female patients (25%), a proportion that significantly exceeds the female representation in several major surgical series, including MARS2 (13%) [33]. This representation strengthens the validity of our sex-stratified analyses.

## 5. Conclusions

Collectively, our findings identify a sex-based disparity in documented asbestos exposure histories, revealing that current clinical approaches to characterize asbestos exposure in PM may prioritize occupational exposures and miss non-occupational exposure routes in female patients, not adequately capturing the evolving epidemiological profile of the disease. As the decline in mesothelioma incidence among females continues to lag behind that of males, efforts to improve asbestos exposure assessment should include sex-sensitive approaches that thoroughly assess non-occupational and environmental exposure pathways. Without such an approach, the true burden of disease in females will remain clinically under ascertained.

Furthermore, our findings support further evaluation of PD-L1 and Ki-67 expression for clinical risk stratification. Conversely, our data suggest that BAP1 should be re-evaluated as a therapy-specific predictive factor rather than a universal prognostic biomarker. Importantly, however, further investigation in larger, treatment-specific cohorts is required to confirm these distinctions. Altogether, continuing to integrate routine immunohistochemistry and other personalized medicine approaches into standard clinical risk assessment could offer critical insights, guiding the escalation of care, the timing of surgical intervention, and the precise selection of complementary multimodal therapies.

## Figures and Tables

**Table 1:** Characteristics of surgical mesothelioma patients by sex.

		All 106 (100%)	Female 27 (25%)	Male 79 (75%)	*p*-Value
	Demographics				
Age *years* (SD)	(*n* = 106)	67.5 (11.0)	64.6 (12.1)	68.5 (10.5)	0.150
Asbestos Exposure n (%)	(*n* = 92)				<0.001
*Likely*		66 (72%)	9 (41%)	57 (81%)	
*No*		26 (28%)	13 (59%)	13 (19%)	
Exposure Source n (%)	(*n* = 59)				<0.001
*Occupational*		46 (78%)	2 (25%)	44 (86%)	
*Non-occupational*		13 (22%)	6 (75%)	7 (14%)	
	Tumor Characteristics and Key Biomarkers				
Histotype *n* (%)	(*n* = 106)				0.471
*Epithelioid*		86 (81%)	24 (89%)	62 (78%)	
*Biphasic*		16 (15%)	2 (7%)	14 (18%)	
*Sarcomatoid*		4 (4%)	1 (4%)	3 (4%)	
BAP1 Expression	(*n* = 81)				1.000
*Intact*		35 (43%)	10 (45%)	25 (42%)	
*Loss*		46 (57%)	12 (55%)	34 (58%)	
PD-L1 TPS	(*n* = 68)				0.080
≤*15%*		50 (74%)	12 (57%)	38 (81%)	
>15%		18 (26%)	9 (43%)	9 (19%)	
Ki-67/MIB-1	(*n* = 49)				1.000
≤*15%*		19 (39%)	3 (33%)	16 (40%)	
>15%		30 (61%)	6 (67%)	24 (60%)	

Values are reported as mean (SD) or *n* (%) for numerical and categorical variables, respectively. Percentages in the ALL column reflect the proportion among patients with available data for each variable; percentages in the Female and Male columns reflect the proportion within each sex group. A two-sample *t*-test (Age) or Fisher’s exact test (Asbestos exposure, Exposure source, Histotype, BAP1 expression, PD-L1 TPS, and Ki-67/MIB-1) was performed to determine significance. Sub-analysis for occupational vs. non-occupational exposure was conducted on patients with a likely exposure history who had a classifiable exposure source (*n* = 59). Missing data: asbestos exposure *n* = 14; exposure source *n* = 7 of likely exposures; BAP1 expression *n* = 25; PD-L1 TPS *n* = 38; KI-67/MIB-1 *n* = 57.

**Table 2:** Independent predictors of likely asbestos exposure.

	Univariable OR (95% CI)	*p*-Value	Multivariable OR_adj_ (95% CI)	*p*-Value
Sex				
*Male vs. female*	6.33 (2.28–18.60)	<0.001	5.45 (1.90–16.35)	0.002
Age *(per year)*	1.04 (1.01–1.09)	0.031	1.04 (0.99–1.08)	0.110
Histotype				
*Epithelioid*	Ref.	-	Ref.	-
*Sarcomatoid*	1.25 (0.15–25.93)	0.853	1.80 (0.13–63.24)	0.700
*Biphasic*	1.38 (0.38–6.61)	0.645	1.24 (0.30–6.60)	0.781

OR = odds ratio; OR_adj_ = adjusted odds ratio; CI = confidence interval. Univariable and multivariable logistic regression models were used to calculate the odds of “Likely Exposure” (*n* = 66) compared to “No Exposure” (*n* = 26). The multivariable model (*n* = 92) was adjusted for age, sex, and histological subtype to determine independent associations. An OR > 1.0 indicates an increased likelihood of exposure. Missing data: asbestos exposure *n* = 14, excluded from the regression analysis.

## Data Availability

The data presented in this study are available on request from the corresponding author. The data are not publicly available due to institutional policies and patient privacy considerations.

## Supplementary Materials

The following supporting information can be downloaded at https://www.mdpi.com/article/10.3390/cancers18162624/s1.

## Abbreviations

The following abbreviations are used in this manuscript:

PM	Pleural mesothelioma
PD-L1	Programmed death-ligand 1
BAP1	BRCA1-associated protein 1
P/D	Pleurectomy/decortication
eP/D	Extended pleurectomy/decortication
EMR	Electronic medical record
SD	Standard deviation
CI	Confidence interval
IQR	Interquartile range
OR	Odds ratio
OR_adj_	Adjusted odds ratio
HR	Hazards ratio
HR_adj_	Adjusted hazards ratio
