# Supplementary material for: Sex-Specific Patterns of Asbestos Exposure and Prognostic Biomarkers in Surgically Treated Pleural Mesothelioma: A Retrospective Cohort Study
Source: Cancers (Basel). 2026 Aug 14;18(16):2624. doi: 10.3390/cancers18162624 (PMC13511244; doi:10.3390/cancers18162624)
Supplement: Supplementary file 1 [file cancers-18-02624-s001.zip › cancers-4427569-supplementary.pdf]

Supplementary Table S1. Sensitivity analysis excluding the inferred occupational exposure category (category 5): characteristics of surgical mesothelioma patients by sex.

		**All**<br>99 (100%)	**Female**<br>27 (27%)	**Male**<br>72 (73%)	**p-value**
Demographics					
Age *years* (SD)	(n=99)	67.0 (11.1)	64.6 (12.1)	67.8 (10.6)	0.233
Asbestos Exposure n (%)	(n=85)				0.002
Likely		59 (69%)	9 (41%)	50 (79%)	
No		26 (31%)	13 (59%)	13 (21%)	
Exposure Source n (%)	(n=52)				<0.001
Occupational		40 (77%)	2 (25%)	38 (86%)	
Non-occupational		12 (23%)	6 (75%)	6 (14%)	

Values are reported as mean (SD) or n (%) for numerical and categorical variables, respectively. Percentages in the ALL column reflect the proportion among patients with available data for each variable; percentages in the Female and Male columns reflect the proportion within each sex group. A two-sample t-test (Age) or Fisher's exact test (Asbestos exposure, Exposure source) was performed to determine significance. Sub-analysis for occupational vs. non-occupational exposure was conducted on patients with a likely exposure history who had a classifiable exposure source (n=52). Missing data: asbestos exposure n=14; exposure source n=7 of likely exposures.

Supplementary Table S2. Sensitivity analysis excluding the inferred occupational exposure category (category 5): independent predictors of likely asbestos exposure.

	**Univariable OR** (95% CI)	**p-value**	**Multivariable OR_{adj}** (95% CI)	**p-value**
Sex				
Male vs female	5.56 (1.99-16.38)	<0.001	4.99 (1.74-14.92)	0.003
Age *(per year)*	1.04 (1.00-1.08)	0.061	1.03 (0.99-1.08)	0.175

OR = odds ratio; OR_{adj} = adjusted odds ratio; CI = confidence interval. Univariable and multivariable logistic regression models were used to calculate the odds of "Likely Exposure" (n=59) compared to "No Exposure" (n=26). The multivariable model (n=85) was adjusted for age and sex to determine independent associations. An OR > 1.0 indicates an increased likelihood of exposure. Missing data: asbestos exposure n=14, excluded from the regression analysis.

Supplementary Table S3. Comparison of patient characteristics between patients with documented versus missing/unknown asbestos exposure data.

		**All**<br>106 (100%)	**Documented**<br>92 (87%)	**Missing/Unknown**<br>14 (13%)	**p-value**
Demographics					
Age *years* (SD)	(n=106)	67.5 (11.0)	67.5 (11.5)	67.5 (7.42)	0.996
Sex n (%)	(n=106)				0.341
Female		27 (26%)	22 (24%)	5 (36%)	
Male		79 (74%)	70 (76%)	9 (64%)	
Tumor Characteristics					
Histotype n (%)	(n=106)				0.695
Epithelioid		86 (81%)	75 (82%)	11 (79%)	
Biphasic		16 (15%)	13 (14%)	3 (21%)	
Sarcomatoid		4 (4%)	4 (4%)	0 (0%)	

Values are reported as mean (SD) or n (%) for numerical and categorical variables, respectively. A two-sample t-test (Age) or Fisher's exact test (Sex, Histotype) were used to determine significance. "Missing/unknown" reflects patients with no documented asbestos exposure history in the EMR (n=14).

Supplementary Table S4. Sensitivity analysis of multivariable Cox proportional hazards models for overall survival according to alternative PD-L1 tumor proportion score cutoffs.

PD-L1 cutoff	HR_{adj} (95% CI)	p-value
> 15% (original)	2.80 (1.39 – 5.64)	0.004
≥ 50%	2.58 (1.18 – 5.66)	0.018
> 1%	1.47 (0.75 – 2.87)	0.263

HR_{adj} = adjusted hazard ratio; CI = confidence interval. Separate multivariable Cox proportional hazards models were constructed using alternative PD-L1 tumor proportion score cutoffs (>1% and ≥50%). Each model was adjusted for age, sex, and histological subtype to determine the independent association between PD-L1 expression and overall survival. An HRadj > 1.0 indicates an increased risk of mortality. Survival data were obtained from electronic medical records and supplemented with publicly available obituary data to minimize loss to follow-up through Jan 15, 2026. All models included 68 patients with available PD-L1 data.

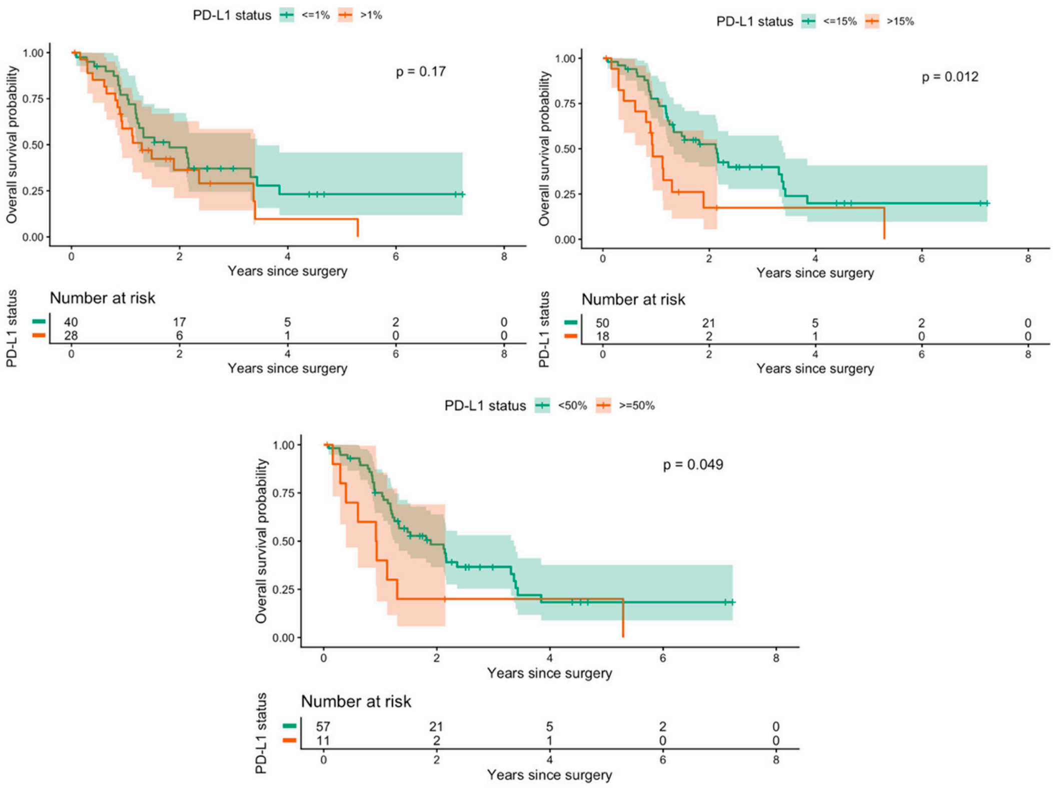

Supplementary Figure S1. Sensitivity analysis of overall survival according to alternative PD-L1 tumor proportion score cutoffs. Kaplan-Meier survival curves illustrating overall survival of patients undergoing surgical intervention for pleural mesothelioma stratified by PD-L1 tumor proportion score using alternative cutoffs: **(A)** ≤ 1% vs. > 1%, **(B)** ≤ 15% vs. > 15%, and **(C)** < 50% vs. ≥50%. Survival data were obtained from electronic medical records and supplemented with publicly available obituary data to minimize loss to follow-up through Jan 15, 2026. Shaded areas represent 95% confidence intervals. A log-rank test was performed to determine p-values and significance. The number of patients at risk is provided below each panel. *N = 68* for all panels.

Supplementary Table S5. Comparison of key characteristics between patients with documented versus missing BAP1 biomarker data.

		All<br>106 (100%)	Documented<br>81 (76%)	Missing<br>25 (24%)	p-value
			Demographics		
Age *years* (SD)	(n=106)	67.5 (11.0)	67.2 (11.2)	68.3 (10.6)	0.676
Sex n (%)	(n=106)				0.649
Female		27 (26%)	22 (27%)	5 (20%)	
Male		79 (74%)	59 (73%)	20 (80%)	
			Tumor Characteristics		
Histotype n (%)	(n=106)				0.638
Epithelioid		86 (81%)	64 (79%)	22 (88%)	
Biphasic		16 (15%)	13 (16%)	3 (12%)	
Sarcomatoid		4 (4%)	4 (5%)	0 (0%)	
			Survival		
Overall survival			57 events	19 events	0.221

Values are reported as mean (SD) or n (%) for numerical and categorical variables, respectively. A two-sample t-test (Age) or Fisher's exact test (Sex, Histotype) were used to determine significance. Overall survival was compared between groups using a log-rank test (χ^2=1.5, df=1). "Missing" reflects patients without BAP1 immunohistochemistry results available in the EMR (n=25).

Supplementary Table S6. Comparison of key characteristics between patients with documented versus missing PD-L1 biomarker data.

		**All**<br>106 (100%)	**Documented**<br>68 (64%)	**Missing**<br>38 (36%)	**p-value**
Demographics					
Age *years* (SD)	(n=106)	67.5 (11.0)	67.1 (11.1)	68.3 (11.0)	0.590
Sex n (%)	(n=106)				0.139
Female		27 (26%)	21 (31%)	6 (16%)	
Male		79 (74%)	47 (69%)	32 (84%)	
Tumor Characteristics					
Histotype n (%)	(n=106)				1.000
Epithelioid		86 (81%)	55 (81%)	31 (81%)	
Biphasic		16 (15%)	10 (15%)	6 (16%)	
Sarcomatoid		4 (4%)	3 (4%)	1 (3%)	
Survival					
Overall survival			47 events	29 events	0.343

Values are reported as mean (SD) or n (%) for numerical and categorical variables, respectively. A two-sample t-test (Age) or Fisher's exact test (Sex, Histotype) were used to determine significance. Overall survival was compared between groups using a log-rank test (χ^2=0.9, df=1). "Missing" reflects patients without PD-L1 immunohistochemistry results available in the EMR (n=38).

Supplementary Table S7. Comparison of key characteristics between patients with documented versus missing Ki-67 biomarker data.

		All 106 (100%)	Documented 49 (46%)	Missing 57 (54%)	p-value
Demographics					
Age *years* (SD)	(n=106)	67.5 (11.0)	67.1 (11.1)	67.8 (11.0)	0.764
Sex n (%)	(n=106)				0.183
Female		27 (26%)	9 (18%)	18 (32%)	
Male		79 (74%)	40 (82%)	39 (68%)	
Tumor Characteristics					
Histotype n (%)	(n=106)				0.607
Epithelioid		86 (81%)	39 (80%)	47 (82%)	
Biphasic		16 (15%)	9 (18%)	7 (13%)	
Sarcomatoid		4 (4%)	1 (2%)	3 (5%)	
Survival					
Overall survival			35 events	41 events	0.317

Values are reported as mean (SD) or n (%) for numerical and categorical variables, respectively. A two-sample t-test (Age) or Fisher's exact test (Sex, Histotype) were used to determine significance. Overall survival was compared between groups using a log-rank test (χ^2=1.0, df=1). "Missing" reflects patients without Ki-67 immunohistochemistry results available in the EMR (n=57).

Supplementary Table S8. Sensitivity analysis of multivariable Cox proportional hazards models for overall survival restricted to epithelioid histology (n=86).

Variable	HR (95% CI)	p-value
BAP1 (Loss vs. Intact)	1.08 (0.57-2.04)	0.814
PD-L1 (>15% vs. <= 15%)	3.81 (1.71-8.47)	<0.001
Ki-67 (>15% vs. <= 15%)	5.35 (2.05-13.98)	<0.001

HR_{adj} = adjusted hazard ratio; CI = confidence interval. Separate multivariable Cox proportional hazards models were constructed restricted to patients with epithelioid histology (n=86). Each model was adjusted for age and sex. An HRadj > 1.0 indicates an increased risk of mortality. Survival data were obtained from electronic medical records and supplemented with publicly available obituary data to minimize loss to follow-up through Jan 15, 2026. Models included 64 patients for BAP1 (42 events), 55 patients for PD-L1 (35 events), and 39 patients for Ki-67 (25 events).
